# Surveillance for SARS-CoV-2 in Norway Rats (*Rattus norvegicus*) from Southern Ontario

**DOI:** 10.1155/2023/7631611

**Published:** 2023-05-26

**Authors:** Sarah J. Robinson, Jonathon D. Kotwa, Simon P. Jeeves, Chelsea G. Himsworth, David L. Pearl, J. Scott Weese, L. Robbin Lindsay, Antonia Dibernardo, Nikki P. L. Toledo, Bradley S. Pickering, Melissa Goolia, Hsien-Yao Chee, Juliette Blais-Savoie, Emily Chien, Winfield Yim, Lily Yip, Samira Mubareka, Claire M. Jardine

**Affiliations:** ^1^Department of Pathobiology, Ontario Veterinary College, University of Guelph, Guelph, Ontario, Canada; ^2^Sunnybrook Research Institute, Toronto, Ontario, Canada; ^3^School of Population and Public Health, University of British Columbia, Vancouver, British Columbia, Canada; ^4^Department of Population Medicine, Ontario Veterinary College, University of Guelph, Guelph, Ontario, Canada; ^5^Public Health Agency of Canada, National Microbiology Laboratory, Winnipeg, Manitoba, Canada; ^6^National Centre for Foreign Animal Disease, Canadian Food Inspection Agency, Winnipeg, Manitoba, Canada; ^7^Department of Medical Microbiology and Infectious Diseases, University of Manitoba, Winnipeg, Manitoba, Canada; ^8^Department of Veterinary Microbiology and Preventative Medicine, College of Veterinary Medicine, Iowa State University, Ames, Iowa, USA; ^9^Global Health Research Center and Division of Natural and Applied Sciences, Duke Kunshan University, Kunshan, Jiangsu, China; ^10^Faculty of Medicine, University of Toronto, Toronto, Ontario, Canada; ^11^Canadian Wildlife Health Cooperative, Ontario Veterinary College, University of Guelph, Guelph, Ontario, Canada

## Abstract

The emergence of severe acute respiratory syndrome coronavirus 2 (SARS-CoV-2) from wildlife has raised concerns about spillover from humans to animals, the establishment of novel wildlife reservoirs, and the potential for future outbreaks caused by variants of wildlife origin. Norway rats (*Rattus norvegicus*) are abundant in urban areas and live in close proximity to humans, providing the opportunity for spillover of SARS-CoV-2. Evidence of SARS-CoV-2 infection and exposure has been reported in Norway rats. We investigated SARS-CoV-2 infection and exposure in Norway rats from Southern Ontario, Canada. From October 2019 to June 2021, 224 rats were submitted by collaborating pest control companies. The majority of samples were collected in Windsor (79.9%; *n* = 179), Hamilton (13.8%; *n* = 31), and the Greater Toronto Area (5.8%; *n* = 13). Overall, 50.0% (*n* = 112) were female and most rats were sexually mature (55.8%; *n* = 125). Notably, 202 samples were collected prior to the emergence of variants of concern (VOC) and 22 were collected while the Alpha variant (B.1.1.7) was the predominant circulating VOC in humans. Nasal turbinate (*n* = 164) and small intestinal (*n* = 213) tissue samples were analyzed for SARS-CoV-2 RNA by RT-PCR. Thoracic cavity fluid samples (*n* = 213) were tested for neutralizing antibodies using a surrogate virus neutralization test (sVNT) (GenScript cPass); confirmatory plaque reduction neutralization test (PRNT) was conducted on presumptive positive samples. We did not detect SARS-CoV-2 RNA in any samples tested. Two out of eleven samples positive on sVNT had neutralizing antibodies confirmed positive by PRNT (1 : 40 and 1 : 320 PRNT70); both were collected prior to the emergence of VOC. It is imperative that efforts to control and monitor SARS-CoV-2 include surveillance of rats and other relevant wildlife species as novel variants continue to emerge.

## 1. Introduction

Severe acute respiratory syndrome coronavirus 2 (SARS-CoV-2) in humans originated from a wildlife host [1] and subsequent human-animal transmission has been noted internationally, involving a range of animal species. Given the zoonotic origins, there are concerns about the establishment of novel animal reservoirs. This could make controlling outbreaks in humans more difficult and provide an opportunity for the development of new variants in animal hosts. Based on evidence from both *in silico* modeling of angiotensin-converting enzyme 2 (ACE2) receptor-binding domain and experimental studies, several animal species are susceptible to SARS-CoV-2 [2–4], and natural SARS-CoV-2 infection has been reported in captive and noncaptive animals including American mink (*Neogale vison*), dogs (*Canis familiaris*), cats (*Felis catus*), tigers (*Panthera tigris*), lions (*Panthera leo*), commercial hamsters, and white-tailed deer (*Odocoileus virginianus*) [5–10].

Although human-to-human SARS-CoV-2 transmission has been the primary driver of the COVID-19 pandemic, limited animal-to-human transmission has occurred from farmed mink, a domestic cat, and commercial hamsters [6, 9, 11, 12]. A suspected instance of wild deer-to-human transmission has also been described [10]. Identifying potential animal reservoirs of SARS-CoV-2 is essential for risk assessment, informing disease control strategies, and for preventing the development of new variants in nonhuman animal hosts. Thus, the surveillance of key captive and wildlife species, particularly at the human-animal interface, is critical.

Surveillance in wildlife across Canada has been ongoing with an emphasis on a One Health approach, a transdisciplinary approach that includes key stakeholders in human and animal health. In a recent study by Greenhorn et al. [13], SARS-CoV-2 was not detected in a sample of 776 animals, including 17 wildlife species from Ontario and Quebec, including raccoons (*Procyon lotor*), skunks (*Mephitis mephitis*), American mink (*Neogale vison*), and big brown bats (*Eptesicus fuscus*). More recently, screening efforts detected the first instance of SARS-CoV-2 in Canadian wildlife in white-tailed deer in Quebec [7]; follow-up whole genome sequencing indicated that the deer were infected with the Delta (B.1.617.2) variant, reflecting contemporaneous VOC activity in proximal human populations. Moreover, highly divergent SARS-CoV-2 was detected in Ontario deer, suggesting circulation within this host population over a protracted period of time [10].

Norway rats (*Rattus norvegicus*) are peridomestic wildlife species (i.e., species that live in close proximity to humans) that exist at the human-wildlife interface providing the opportunity for spillover of zoonotic pathogens from humans to wildlife. Notably, evidence of SARS-CoV-2 infection and exposure have been reported in peridomestic Norway rats [14, 15]. Cryptically circulating SARS-CoV-2 lineages (i.e., lineages previously undetected by surveillance systems and not recognized in the GISAID database) have been detected in New York City wastewater, which may indicate the presence of animal reservoirs in the urban environment [16]. According to experimental studies and ACE2 receptor binding analysis, the susceptibility of rats to ancestral SARS-CoV-2 is unclear [17–20]. However, as new variants emerge, the host range may also change, as demonstrated by the susceptibility of rats to the Alpha (B.1.1.7), Delta, and Omicron (B.1.1.529) variants [15, 19].

We assessed infection with and exposure to SARS-CoV-2 in peridomestic Norway rats (*Rattus norvegicus*) from regions in Southern Ontario, focusing on cities.

## 2. Materials and Methods

### 2.1. Sample Collection

Rat carcasses were obtained through collaboration with pest control professionals working in regions of Southern Ontario with a focus on urban areas, including Hamilton, Windsor, and the Greater Toronto Area (i.e., Halton, Peel, City of Toronto, and Durham regions). Between October 1, 2019, and June 22, 2021, pest control technicians submitted carcasses collected during regular pest control activities and delivered carcasses to a designated location for freezing and storage. No animals were killed for the purpose of this study. Rats were submitted with the date and geographic location of collection. Carcasses were stored at −24°C and shipped to the University of Guelph, Guelph, Ontario, for full necropsy. Tissue samples were collected aseptically (i.e., nasal turbinate and small intestine stored at −80°C) for molecular testing and thoracic cavity fluid on Nobuto filter strips stored at 4°C (Advantec MFS, Inc., Dublin, CA, USA, and Fisher Scientific, Waltham, MA, USA) under biosafety level 2 (BSL-2) conditions with institutional approval; not all sample types were available in all instances due to poor carcass condition or sampling protocol. Necropsies performed before March 2020 did not include collection of nasal turbinate samples. During necropsy, species (external morphology), sex, and sexual maturity (open vaginal orifice in females and scrotal testes in males) were recorded.

### 2.2. RNA Extraction and RT-PCR

RNA extraction and reverse transcription-polymerase chain reaction (RT-PCR) testing were performed at the Sunnybrook Research Institute in Toronto, Ontario, under BSL-2+ with institutional approval. Tissue samples were thawed, weighed, minced with a scalpel, and homogenized in 600 *μ*L of NucliSens Lysis Buffer spiked with armored RNA enterovirus (Asuragen; https://www.asuragen.com) using the next advance bullet blender (Next Advance, Troy, NY, USA) and a 5 mm stainless steel bead at 5 m/s for 4 minutes. RNA from 30 mg tissue samples was extracted via the NucliSens easyMAG using Specific Protocol B 2.0.1; RNA was eluted in 50 *μ*L. All extractions were performed with a negative control.

Reverse transcription-polymerase chain reaction was performed as previously described [13]. In brief, two gene targets were used for SARS-CoV-2 RNA detection: the 5' untranslated region (UTR) and the envelope (E) gene [21]. A positive control, negative extraction control, and no-template control were included in each analysis. All samples were run in duplicate, and samples with cycle thresholds <40 for both gene target and armored RNA enterovirus in at least one replicate were considered positive.

### 2.3. Serology

The collection of suitable specimens from carcasses of varying quality for serological testing is challenging; the availability of serum and/or plasma samples, suitable for analysis, is limited since changes that occur postmortem typically reduce the quantity and quality of liquid blood [22]. As such, we relied on the collection of thoracic cavity fluid (i.e., a mixture of blood and fluid exudate) on Nobuto filter strips as a serological specimen for analysis. Antibody testing was performed at the National Microbiology Laboratory in Winnipeg, Manitoba. All samples were stored at 4°C prior to testing. Thoracic cavity fluid samples were collected onto Nobuto filter strips (Advantec MFS, Inc., Dublin, CA, USA and Fisher Scientific, Waltham, MA, USA) by saturating the length of the strip with 100 *μ*l of fluid. To obtain the 1 : 9 dilution required for testing, saturated Nobuto filter strips were cut into 4-5 pieces and placed in a 2 ml tube containing 360 *μ*l phosphate-buffered saline (PBS) pH 7.4 and eluted overnight at 4°C. Samples were mixed by vortexing and tested using a surrogate viral neutralization test (sVNT), GenScript cPass™ SARS-CoV-2 Neutralization Antibody Detection Kit (GenScript USA, Inc., Piscataway, NJ, USA) according to the manufacturer's protocol. In brief, 60 *μ*l of a sample was added to 60 *μ*l HRP-conjugated RBD solution and incubated at 37°C for 30 minutes. A 100 *μ*l aliquot of the mixture was transferred to the ELISA microwell test plate and incubated at 37°C for 15 minutes. Microwells were washed 4 times with 260 *μ*l wash buffer, then 100 *μ*l TMB substrate was added to each well. Following a 20-minute incubation in the dark at room temperature, 50 *μ*l of stop solution was added to each well. Absorbance was read immediately at 450 nm.

Each assay plate included positive and negative controls provided by the manufacturer that met required quality control parameters. In addition, in-house controls consisting of human serum samples that were known to be positive and negative for SARS-CoV-2 antibodies were included in each run. Percentage inhibition was calculated for each sample using the following equation: percent inhibition = (1 − optical density sample/optical density negative control) × 100%. Samples with greater than or equal to 30% inhibition were considered presumptive positive for SARS-CoV-2-neutralizing antibodies.

Confirmatory plaque reduction neutralization test (PRNT) testing for sVNT positive samples was performed in the Zoonotic BSL-3 facility at the CFIA using ancestral SARS-CoV-2 (SARS-CoV-2/Canada/VIDO-01/2020) in Vero E6 cells. Nobuto strips were prepared by elution in 400 *μ*L DMEM containing 1% BSA and 1% Pen/strep and heat inactivated at 56°C for 30 minutes. Eluates were serially diluted two fold and incubated with challenge virus at a concentration to produce 100 PFU/well. Nobuto eluates/virus mixture was incubated at 37°C, 5% CO_2_ for 1 hour. The mixture was then added to 24-hour cultures of Vero E6 cells that were previously seeded in 48-well plates and further incubated at 37°C, 5% CO_2_ for 1 hr. The mixture was removed and 0.5 mL of 1.5% carboxymethylcellulose (CMC) salt overlay was added to each well. Plates were incubated at 37°C, 5% CO_2_ for 72 hours. Two fold dilutions of the virus without antibody and known SARS-CoV-2 positive and negative Nobuto eluates were used as controls in this assay. Plates were fixed with 10% formalin, and plaques were visualized by staining with 0.5% crystal violet. Antibody titres were defined as the highest serum dilution that resulted in >70% (PRNT70) reduction in the number of plaques. Only rats positive on sVNT and PRNT70 were considered seropositive in this study.

### 2.4. Spatial and Graphic Visualization

Rat collection sites were classified according to land use as defined by Himsworth et al. [23] (i.e., residential, commercial, industrial, institutional, green space, vacant/undeveloped, abandoned, open, and mixed). Rat location data were superimposed on human population density within Southern Ontario by the municipality [24, 25]. Data for Windsor and Hamilton were visualized at a finer geographic resolution and superimposed on human population density by census tract [26, 27]. Graphic displays were produced via QGIS 2.14.3 (Quantum GIS Development Team; http://www.qgis.org). Human coronavirus disease 2019 (COVID-19) data were accessed through Public Health Ontario [28]. Rat collection count, human COVID-19 cases per 100,000 people by date, and rat seropositivity were visualized graphically for the province of Ontario, as well as Windsor and Hamilton using STATA 15 (StataCorp, College Station; http://stata.com).

## 3. Results

### 3.1. Sample Collection

**Figure 1:**
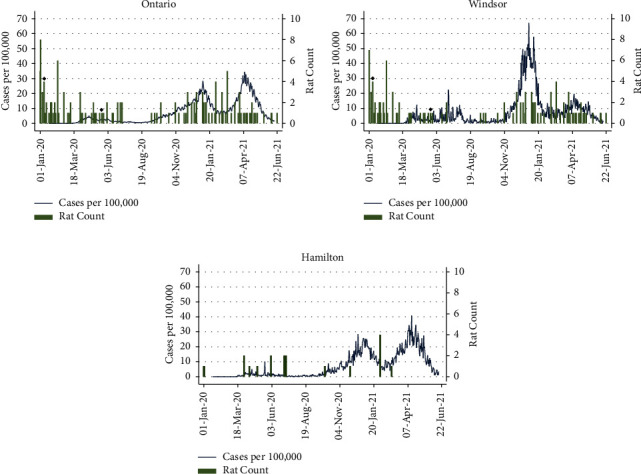
Rat collection counts and human COVID-19 cases per 100,000 people by date from January 1, 2020 to June 22, 2021 in (a) Ontario, (b) Windsor, and (c) Hamilton. Collection dates of seropositive rats (PRNT70) are indicated by black diamonds.

**Figure 2:**
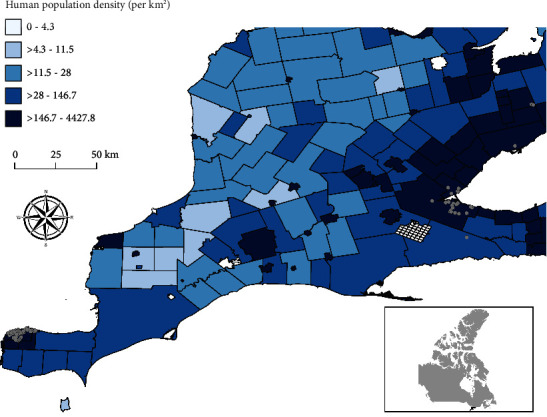
Map of Southern Ontario with the locations of rats collected by pest control companies from October 2019 to June 2021. Collection location data are superimposed on a choropleth map of human population density (per km^2^) by regional county municipalities.

From January 2020 to June 2021, 149 Norway rats were submitted by pest control partners. The temporal distribution of all rats collected for this study compared to the human COVID-19 cases per 100,000 people for the province of Ontario is shown in Figure 1(a). The majority of rats were collected in Windsor (79.9%; *n* = 119) and Hamilton (12.8%; *n* = 19), with some samples from the Greater Toronto Area (6.7%; *n* = 10) and Haldimand County (0.7%; *n* = 1) (Figure 2). Figures 1(b) and 1(c) show the temporal distribution of rat counts in Windsor and Hamilton and human COVID-19 cases per 100,000 people within their respective regions. The number of rats collected during each season varied; the fewest rats were collected in the summer (6.0%; *n* = 9). Rats were collected from residential (61.1%; *n* = 91), institutional (14.8%; *n* = 22), commercial (9.4%; *n* = 14), industrial (8.1%; *n* = 12), and mixed (6.7%; *n* = 10) land use categories. Rats were not collected from green space, open, vacant/undeveloped, or abandoned land. A subset of rats (*n* = 75) from October to December 2019 were also tested as a prepandemic group. These rats were collected primarily in Windsor (80%; *n* = 60) and Hamilton (16%; *n* = 12), with a minority of samples collected in the Greater Toronto Area (4%; *n* = 3). In total, 224 Norway rats were analyzed in this study.

**Figure 3:**
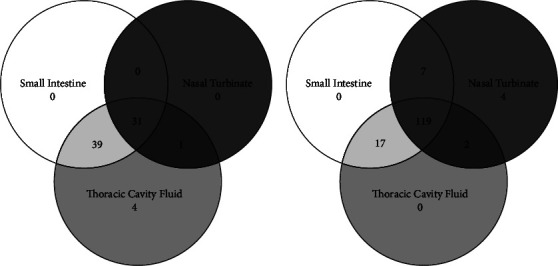
Sample types collected for rats submitted by pest control companies in Southern Ontario for the (a) prepandemic group (*n* = 75) collected between October and December 2019 and the (b) pandemic group (*n* = 149) collected between January 2020 and June 2021.

At necropsy, 164 nasal turbinate, 213 small intestinal tissue samples, and 213 thoracic cavity fluid samples were collected. A breakdown of sample types collected for individual rats in the pandemic and prepandemic groups is shown in Figure 3. All rats were identified as Norway rats based on external morphology. Of the 224 rats, 50.0% (*n* = 112) were female and 45.1% (*n* = 101) were male; most rats were sexually mature (55.8%; *n* = 125). Sex (*n* = 11) and sexual maturity (*n* = 12) could not be determined in some rats due to poor carcass condition.

### 3.2. RT-PCR

SARS-CoV-2 RNA was not detected in any of the 149 rats from the pandemic group (95% confidence interval (CI): 0–3.0%); no evidence of SARS-CoV-2 RNA was detected in rats from the prepandemic group (*n* = 75).

### 3.3. Serology

Neutralizing antibodies against SARS-CoV-2 were detected in 11 thoracic cavity fluid samples (sVNT), two of which were confirmed by PRNT70 (Table 1). The PRNT70 titres were 1 : 40 and 1 : 320. One rat (1 : 40 titre) was a juvenile female collected on January 10, 2020, and the other rat (1 : 320 titre) was an adult female collected on May 20, 2020. Both seropositive rats were collected in Windsor.

## 4. Discussion

We did not find any evidence of SARS-CoV-2 viral RNA in the present study. Similarly, two studies conducted in Europe in late 2020 and from 2020–2022 found no evidence of SARS-CoV-2 infection in rats in Belgium or Germany, respectively [29, 30]. Conversely, one study found evidence of neutralizing antibodies in a Norway rat in Hong Kong in May 2021 [14]. Another study conducted in New York City in September 2021 showed evidence of both exposure and infection [15]. Results from our serological testing indicate two seropositive rats, both were collected from residential sites in Windsor. The two seropositive rats were collected in January 10 and May 20, 2020, when ancestral variants were the predominantly circulating viruses. Interestingly, one of the seropositive rats was collected prior to when the first official known human case of SARS-CoV-2 in Ontario was confirmed January 25, 2020 [31]. Notably, the state of Michigan, which borders Windsor, did not report its first presumptive human case of SARS-CoV-2 until March 10, 2020 [32]. At the time the second rat was collected, Windsor and Essex County had the fourth highest incidence rate in Ontario (189 cases per 100,000 people from March 20 to May 21, 2020), with the majority of cases (60%) in the City of Windsor [33].

Based on results from receptor binding analyses and experimental challenge studies, the evidence for rat susceptibility to ancestral SARS-CoV-2 is conflicting [19, 20]; however, rats are susceptible to several variants (e.g., Alpha, Delta, and Omicron) [15, 17, 18]. It is interesting that the two seropositive rats were collected before the reported emergence of variants, starting in late 2020. It is likely that the detected seropositive rats were exposed to the ancestral SARS-CoV-2; however, exact identification of the SARS-CoV-2 that the rats were exposed to is not possible. Although no other study has identified exposure of SARS-CoV-2 in Norway rats prior to the emergence of variants, surveillance for SARS-CoV-2 in rats has been limited. It should be noted that the previous susceptibility work for rats with SARS-CoV-2 is based on challenge studies and conducted on a small number of young, healthy individuals, which may not be representative of the full breadth of possible responses to infection in the wild.

It is unclear how these rats may have been exposed to SARS-CoV-2. Since rats are peridomestic species and thrive in anthropogenic settings, exposure to the virus could occur through direct or indirect contact with humans and human contaminated environments as previously hypothesized [3, 34, 35]. In addition, SARS-CoV-2 is shed in human feces, and viral RNA has been detected in wastewater and urban run-off, which could represent a potential route of exposure to wildlife species [36, 37]. To date, however, there is no conclusive evidence of infectious virus in wastewater [38].

Serological assays can be subject to cross-reactivity. The previous work has shown little evidence for cross-reactivity with other serological assays (e.g., GenScript cPass sVNT) and other alpha and beta coronaviruses in several species (e.g., dogs, cats, and hamsters); based on sera from multiple species, the sVNT has a sensitivity of 98.9% and specificity of 98.8% [39]. However, we cannot rule out the potential of cross-reactivity with an endemic coronavirus circulating in the sampled rat population. While a limited number of studies have detected coronaviruses in rat species in some areas of the world [40–42], there is a paucity of knowledge on coronaviruses in rat populations in Canada. In order to rule out false positives due to cross-reactive antibodies, we employed a follow-up confirmatory PRNT, which is considered the gold standard for antibody detection for viral infections [43]. Perera et al. [39] found that PRNT was marginally less sensitive than sVNT for antibody detection in confirmed SARS-CoV-2 infection in humans.

From a One Health perspective, the establishment of an animal reservoir is of particular concern due to the potential development and emergence of novel variants of wildlife origin. It has been recently proposed that Omicron may have originated in mice based on significant overlap in mutations in the spike protein with SARS-CoV-2 mutations known to promote adaptations in mouse hosts [44]. In addition, since we did not detect viral RNA, we could not assess possible pathology associated with infection, so the implications for rat health are unknown. Maintaining relationships with the industry and government partners for continuous longitudinal and targeted surveillance in rodents and other relevant wildlife species at the human-animal interface is pertinent. In particular, using a combined PCR and serological screening approach can enhance our understanding of present and past circulation of SARS-CoV-2 in wildlife; PCR is indicative of active or recent infection whereas serological testing provides insights related to exposure or past infection. These data may help differentiate sustained versus unsustained transmission within susceptible wildlife populations to better understand their suitability as a reservoir host for SARS-CoV-2.

There are several limitations to consider for this study. First, sample collection relied on carcass submission from pest control professionals. While this sampling strategy provided us with rat samples from areas with high human population density, it resulted in variations in sampling effort geographically and over our study period due to seasonal fluctuations in pest control submissions, as well as impacts of the nonpharmaceutical interventions (e.g., work from home) for controlling the COVID-19 pandemic. There are limitations to the spatial and temporal distribution of samples collected from pest control companies [45]. Second, not all rats that were analyzed via PCR in this study had corresponding serology testing (*n* = 11), so the exposure status for these individuals is not known. Lastly, since this study used pest control sourced carcasses, there was potential for delayed freezing after field collection. As such, it is possible that rat carcass quality could impact pathogen testing and result in reduced sensitivity. However, the previous work has indicated that pest control sourced rat carcasses are suitable for PCR testing of tissue samples for viral pathogens (e.g., hepatitis E virus) (S Robinson, unpublished data). We relied on the collection of thoracic cavity fluid on Nobuto filter strips as a serological specimen for the analysis, since quality whole blood was not consistently available. Although this sample type has been shown to be a suitable serological specimen substitute for other pathogens [46, 47], it is unclear how suitable this sample type is for the Norway rats and SARS-CoV-2 serological assays in this study.

We report evidence of past exposure to SARS-CoV-2 in Norway rats in North America. However, no evidence of SARS-CoV-2 RNA was detected. To better understand potential cryptic circulation in Norway rats and other wildlife species, continuous surveillance efforts are needed, particularly at the human-animal interface. A One Health approach to SARS-CoV-2 monitoring in wildlife is paramount for the detection of spillover events and monitoring the establishment of wildlife reservoirs.

## Figures and Tables

**Table 1:** Results of confirmatory plaque reduction neutralization testing (PRNT) for the 11 presumptive surrogate virus neutralization test positive thoracic cavity fluid samples collected from rats submitted by pest control companies in Southern Ontario.

Rat identification no.	Date of collection	City	Land use category	PRNT70 titre
277	17-Oct-19	Hamilton	Mixed	—
173	5-Dec-19	Windsor	Industrial	—
216	10-Dec-19	Windsor	Industrial	—
217	16-Dec-19	Windsor	Industrial	—
231	10-Jan-20	Windsor	Residential	1 : 40
188	3-Feb-20	Windsor	Residential	—
230	24-Feb-20	Windsor	Institutional	—
192	1-Apr-20	Windsor	Residential	—
227	20-May-20	Windsor	Residential	1 : 320
251	1-Jul-20	Hamilton	Mixed	—
247	11-Nov-20	Burlington	Residential	—

## Data Availability

The data that support the findings of this study are available from the corresponding author on request.
